# Cardiometabolic and Skeletal Risk Factors in Black Men with Prostate Cancer Starting Androgen Deprivation Therapy

**DOI:** 10.3390/cancers7020679

**Published:** 2015-04-22

**Authors:** Orvar Gunnarsson, Shehzad Basaria, Gretchen A. Gignac

**Affiliations:** 1 Department of Medicine, Division of Hematology and Oncology, Hospital of the University of Pennsylvania, 3400 Spruce Street, 16 Penn Tower, Philadelphia, PA 19104, USA; 2 Department of Medicine, Section of Men’s Health, Aging and Metabolism, Brigham and Women’s Hospital, Boston, MA 02115, USA; E-Mail: sbasaria@partners.org; 3 Department of Medicine, Section of Hematology and Oncology, Boston University School of Medicine, Boston, MA 02118, USA; E-Mail: Gretchen.Gignac@bmc.org

**Keywords:** prostate cancer, androgen deprivation therapy, metabolic complications

## Abstract

*Background*: Androgen deprivation therapy (ADT) for prostate cancer (PCa) is associated with multiple metabolic complications, previously predominantly evaluated in the white population. *Methods*: A chart-based retrospective review was conducted on black patients with PCa, considered for ADT, from September 2007 to July 2010. Baseline data were collected on body mass index (BMI), vitamin-D status, bone mineral density (BMD), dyslipidemia and diabetes. Overweight and obesity were classified as BMI ≥ 25 and BMI ≥ 30, respectively. Vitamin-D sufficiency was defined as levels ≥30 ng/mL, insufficiency as <30 ng/mL and deficiency as ≤20 ng/mL. Osteopenia was defined as T scores between −1 to −2.5 and osteoporosis when T scores ≤−2.5. *Results*: Of the initial cohort of 130 black men, 111 (85.4%) patients underwent ADT. At baseline, average BMI was 28.1 ± 5.9 with 43.3% of men being overweight and 30.8% obese. More than one-third of the patients had pre-existing dyslipidemia while 28.8% were diabetics. 50% were vitamin-D deficient while 41% had low bone mass. *Conclusions*: Black men with PCa presenting for consideration of ADT have a high prevalence of existing metabolic risk factors. Close monitoring of this patient population is needed during ADT to prevent and treat metabolic complications.

## 1. Introduction

Prostate cancer is the most common cancer among men, with an estimated 238,590 new cases diagnosed and 29,720 deaths in 2013 alone, making it the second most common cause of cancer- related deaths after lung cancer [1]. Androgen deprivation therapy (ADT) is the cornerstone of therapy for locally advanced and metastatic prostate cancer where its use has been associated with a survival advantage. However, its use is also associated with numerous adverse effects. In addition to vasomotor symptoms, sexual dysfunction and poor quality of life, ADT can also lead to metabolic complications such as insulin resistance [2,3,4], diabetes [3,5], dyslipidemia [4,6], and metabolic syndrome [3]. These complications are a consequence of body composition changes that are encountered as a result of profound hypogonadism resulting in an increase in fat mass and BMI, and a decrease in lean body mass [7]. These metabolic changes may contribute to the increased cardiovascular disease seen in these patients [5]. Indeed, cardiovascular disease has become the most common cause of death in patients with prostate cancer [8]. Furthermore, patients undergoing ADT also lose bone mass that results in a higher incidence of fractures in these men [9].

Previous studies documenting aforementioned side effects have been conducted on a predominantly Caucasian population [2,3,6,7,9,10,11,12,13,14,15,16]. Far fewer studies have been performed in a racially mixed population [11,17] and only a single previous study evaluated black patients exclusively [18] and then only bone mineral density (BMD). This knowledge gap is concerning as the incidence [19] and mortality [20] of prostate cancer is highest among the black population. In addition to the above, in the general population, the black population has a higher baseline prevalence of diabetes, hypertension, stroke [21] and vitamin-D deficiency [22] as compared with the Caucasian population [21,23,24,25]. It is therefore important to evaluate the baseline prevalence of these risk factors in black men who are starting ADT as the magnitude of worsening of these parameters on ADT may be higher compared to Caucasian men. This was the objective of our study.

## 2. Materials and Methods

### 2.1. Subjects

This was a cross-sectional study in which all patients with the diagnosis of prostate cancer, presenting to a single provider at a University hospital in the Boston area from September 2007 to July 2010, were examined.

### 2.2. Study Procedures

An extensive chart review was conducted of patients presenting to the oncology clinic for evaluation who were determined to be eligible to start ADT. Patients were considered diabetics if they carried a known diagnosis, were on anti-diabetic medications or had a HbA1c of ≥6.5% [26]. Similarly, a subject was considered hyperlipidemic if there was a known diagnosis and/or he was on anti-lipid medications. Height and weight were measured and body mass index (BMI) calculated (kg/m^2^). Underweight was classified as a BMI < 18.5, normal weight as a BMI between 18.5 and 24.9, overweight if BMI was between 25.0 and 29.9 and obesity as BMI ≥ 30 [27].

Osteoporosis was defined as a T score ≤−2.5 and osteopenia if the T score was between −1 and −2.5 [28] measured by Dual Energy X-ray Absorptiometry (DEXA). DEXA scans performed previously or up to 3 months after the start of ADT were considered (as changes in bone mass on DEXA scans are not seen that early after ADT). Vitamin-D insufficiency and deficiency were defined as levels of 25(OH)D <30 ng/mL and 25(OH)D <20 ng/mL respectively [29].

### 2.3. Ethical Approval

The Institutional Review Board for Boston University Medical Center approved the study.

### 2.4. Data Analysis

Data are presented as mean ± standard deviation and percentage (%) where appropriate. Between group differences were analysed via ANOVA for means. Results were considered significant at p values equal to 0.05. All statistical analysis was performed using the SPSS 17.0 for Windows software (SPSS Inc.: Chicago, IL, USA).

## 3. Results

### 3.1. Baseline Characteristics

**Figure 1:**
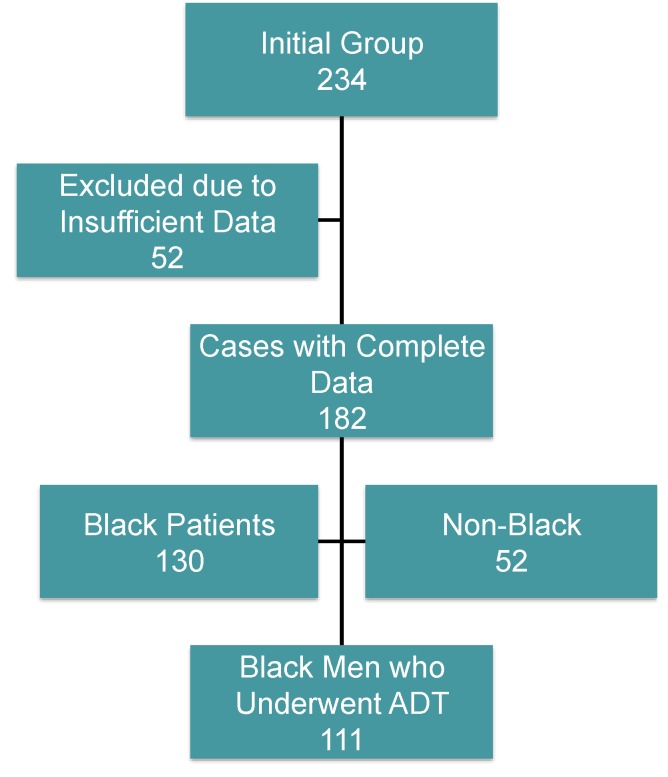
Study profile. Out of the initial study cohort of 234 patients, a total of 182 patients had sufficient data for analysis, of which 130 patients were black and 111 (85.4%) underwent androgen deprivation therapy.

The initial study group consisted of 234 patients, 52 of which did not have sufficient data for analysis. Of the remaining 182 men who had complete data, 52 men were not black, yielding a sample size of 130 black patients. Of these, 111 black men underwent ADT (Figure 1).

**Figure 2:**
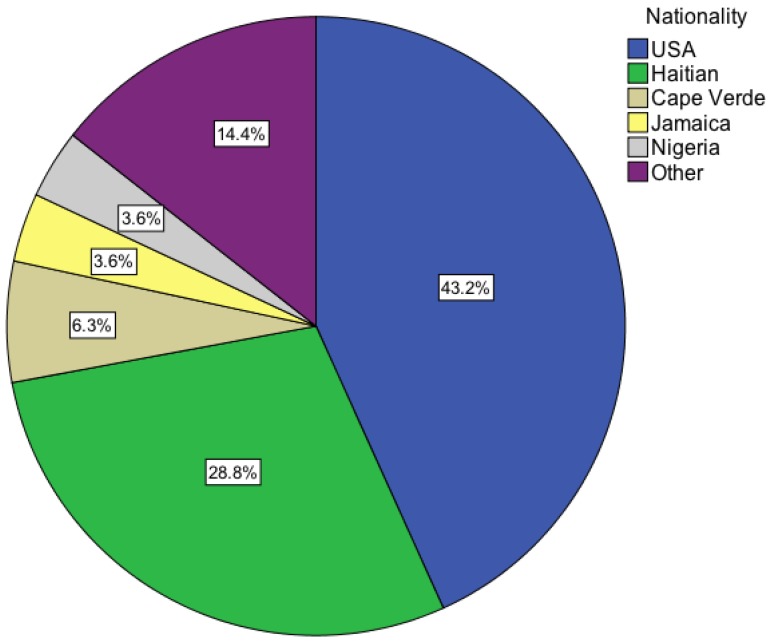
Patient Nationalities. The patients origin was from a total of 16 countries with 43.2% (*n* = 48) being African Americans and 56.8% (*n* = 63) immigrants, of which 52.4% (*n* = 33) came from Haiti and other countries with lower proportions.

This sample of 111 patients was comprised of 48 African-Americans (43.2%) and the remaining 63 patients were black immigrants (56.8%) from 16 countries (Figure 2). The age of the patients ranged from 45 to 92 years (Table 1).

**Table 1:** Baseline patient characteristics. Baseline clinical characteristics of patients in the study population (*n* = 111). ADT = Androgen Deprivation Therapy. PSA = Prostate Specific Antigen. BMI = Body Mass Index. BMD = Bone Mass Density.

Patient characteristics	Mean ± Standard Deviation	Minimum–Maximum
**Age (years)**	68.4 ± 9.0	45–92
**Time to ADT start after Dx(months)**	9.7 ± 19.7	0–108
**Gleason score Total**	7.6 ± 1.0	6–10
**PSA (ng/mL)**	302 ± 790	0.2–5609
**BMI**	28.1 ± 6.0	15.4–58.5
**HbA1c (%)**	6.8 ± 1.2	4.9–10.9
**Total Cholesterol (mg/dL)**	187 ± 53	105–333
**LDL Cholesterol (mg/dL)**	112 ± 39	45–235
**HDL Cholesterol (mg/dL)**	48 ± 16	25–108
**Triglycerides (mg/dL)**	127 ± 66	35–292
**25(OH)D(ng/mL)**	20.5 ± 8.7	4.0–43.0
**BMD—Lumbar spine (T score)**	−0.370 ± 1.464	−4.200–3.340
**BMD—Femoral neck (T score)**	−0.939 ± 1.035	−3.000–1.500

### 3.2. BMI

The average BMI at baseline was 28.1 ± 6.0. Of these, 24 patients (23.1%) were of normal weight (Table 2). A total of 77 patients (74.1%) were either overweight (43.3%) or obese (30.8%). 3 men (2.9%) were considered underweight while anthropometry was not available in 7 patients.

**Table 2:** Patient subgroup classification According to BMI, Dyslipidemia, Diabetes, Vitamin D status and BMD.

Patient subgroup	Category	N	%
**BMI weight classification**	BMI Means ± SD *		
	Underweight 16.5 ± 0.9	3	2.9
	Normal weight 22.6 ± 2.0	24	23.1
	Overweight 27.3 ± 1.4	45	43.3
	Class I obesity 31.7 ± 1.5	22	21.2
	Class II obesity 36.6 ± 1.6	6	5.8
	Class III obesity 46.2 ± 8.4	4	3.8
**Diabetes**	Yes	32	28.8
	No	79	71.2
**Dyslipidemia**	Yes	41	36.9
	No	70	63.1
**Vitamin D status**	25(OH)D ng/mL means ± SD *		
	Vitamin D deficiency 13.5 ± 4.6	44	50.0
	Vitamin D insufficiency 24.5 ± 3.1	30	34.1
	Vitamin D sufficiency 34.2 ± 3.9	14	15.9
**Bone mass density**	T-score ± SD *		
	Osteoporosis −3.160 ± 0.673	5	9.3
	Osteopenia −2.026 ± 0.302	17	31.5
	Normal BMD −0.501 ± 0.699	32	59.3

Underweight: BMI ≤ 18.5, Normal weight: BMI 18.5–24.9, Overweight: BMI 25.0–29.9, Obesity: BMI > 30, Class I obesity: BMI 30.0–34.9, Class II obesity: BMI 35.0–39.9, Class III obesity: BMI ≥ 40.0. Vitamin D deficiency: 25(OH)D < 20 ng/mL, Vitamin D insufficiency 20–30 ng/mL, Vitamin D sufficiency > 30 ng/mL. Osteoporosis: T-score < −2.5, Osteopenia: T-score −1.0–−2.5, Normal BMD: T-score >−1.0. * *p* < 0.001 for within group difference. ** *p* = 0.002 for difference between osteoporosis and osteopenia. BMI = Body Mass Index. BMD = Bone Mass Density.

### 3.3. Diabetes and Hyperlipidemia

Diabetes was present in more than a quarter of the sample (32 men, 28.8%; Table 2). Average baseline HbA1c was 6.8%, which is above the diabetic threshold of 6.5%. Similarly, more than one-third of the patients either carried a diagnosis of hyperlipidemia or were taking lipid lowering medications (41 patients (36.9%)).

### 3.4. Vitamin-D Levels and Bone Mass

Mean vitamin-D levels were 20.5 ng/mL, which is borderline deficient. Only 14 patients (15.9%) were Vitamin-D replete, while 30 (34.1%) had vitamin-D insufficiency levels and 44 (50.0%) were vitamin-D deficient (Table 2). On average, bone mass was low at both the lumbar spine and femoral neck. Of the 54 patients on whom DEXA scans were available, 32 (59.3%) had normal BMD. However, 17 patients (31.5%) were osteopenic and 5 (9.3%) had osteoporosis.

## 4. Discussion

Prior research evaluating the prevalence of metabolic risk factors in prostate cancer patients who were going to start ADT predominantly focused on the Caucasian population. The purpose of our study was to examine the prevalence of these metabolic risk factors in black patients with prostate cancer who were initiating ADT. We found a very high prevalence of both cardiometabolic and skeletal risk factors in this population.

In our study, 74% of patients were either overweight or obese. Previous data show that 68% of black men older than 18 years in the general U.S. population are either obese or overweight. Hence, the prevalence of being overweight and obese was higher in our patients. Other studies have reported 36%–53% prevalence of overweight and 4.3%–44% of obesity [4,13,17]. In our group, mean baseline BMI was 28.1 while other studies have reported BMI ranging from 25.0 to 29.1 [3,4,7,10,13,14]. Since weight gain is routinely seen in men undergoing ADT, the mean BMI of our population is likely to increase during treatment and may even reach the obesity category. It must be emphasized that this weight gain is mainly due to gain in fat mass [7], likely increasing the risk of cardiac disease.

Diabetes prevalence in the U.S. is, in general, significantly more common among African American men compared to Caucasians. For all 65 year old Medicare and Medicaid recipients, the prevalence of diabetes in 2010 was 28.2% [21]. Two prior studies in a racially mixed Veterans Administration population have found prevalence of diabetes of 19.4% [17] and 40% [11] among patients starting ADT. In our cohort, the prevalence of 28.8% starting ADT is significant. Similarly, 37% of our patients were hyperlipidemic. This underscores the significant burden of prevalent metabolic abnormalities in black men starting ADT.

In our group, 40% of patients had either osteopenia or osteoporosis. Furthermore, 74% were either vitamin-D deficient or insufficient. This is higher than previously reported prevalence of osteopenia and osteoporosis of 29% and 5%, respectively [15]. In that study of Caucasian men, only 17% of patients were vitamin-D deficient, underscoring the higher prevalence of vitamin-D deficiency in our cohort. Another study of predominantly Caucasian men showed 18% and 50% prevalence of vitamin D deficiency and insufficiency, respectively [16]. The only study that has been performed on the black population was in Jamaican men that showed 40% of patients had osteopenia while 5% had osteoporosis [18], which is similar to our findings. These observations are meaningful as black men lose bone mass at a similar rate compared to Caucasians [15] and are less likely to get bone density testing [30].

Our study has some limitations. This is a cross-sectional study, and longitudinal studies are needed to evaluate changes in these metabolic parameters in different populations after initiation of ADT. Our study was performed at a single institution, which limits the generalizability of our patient population to other geographic regions and socioeconomic differences. Future studies, in other geographic and socioeconomic settings are needed.

## 5. Conclusions

Patients with prostate cancer, undergoing ADT are at risk for a significant worsening of their underlying metabolic co-morbidities. Prior publications have mainly addressed baseline risk factors such as obesity, dyslipidemia, diabetes, osteoporosis and osteopenia and vitamin-D deficiency and insufficiency in the Caucasian population. Our data shows that these risk factors are highly prevalent among black men with prostate cancer. To our knowledge, this is the first study evaluating these multiple cardiometabolic and skeletal risk factors in black patients with prostate cancer prior to initiating ADT. Our findings should serve to increase awareness of these risk factors among physicians treating this patient population and hopefully highlight the need for preventative measures to take place when indicated and therefore lead to improved patient care for this population.
